# Platelet lysate induces chondrogenic differentiation of umbilical cord-derived mesenchymal stem cells

**DOI:** 10.1186/s11658-018-0080-6

**Published:** 2018-03-20

**Authors:** Ghmkin Hassan, Mohammad Bahjat, Issam Kasem, Chadi Soukkarieh, Majd Aljamali

**Affiliations:** 1 Faculty of Pharmacy, Damascus University, Damascus, Syria; 2 Faculty of Sciences, Damascus University, Damascus, Syria; 3 National Commission for Biotechnology (NCBT), Damascus, Syria

**Keywords:** Platelet lysate, Mesenchymal stem cells, Chondrogenic differentiation, Cartilage

## Abstract

**Purpose:**

Articular cartilage has a poor capacity for self-repair, and thus still presents a major challenge in orthopedics. Mesenchymal stem cells (MSCs) are multipotent stem cells with the potential to differentiate into chondrocytes in the presence of transforming growth factor beta (TGF-β). Platelet lysate (PL) contains a relatively large number of growth factors, including TGF-β, and has been shown to ameliorate cartilage repair. Here, we investigated the ability of PL to direct chondrogenic differentiation of MSCs along with other standard differentiation components in a pellet culture system.

**Methods:**

We isolated and expanded MSCs from human umbilical cords using a PL-supplemented medium and characterized the cells based on immunophenotype and potential for differentiation to adipocytes and osteocytes. We further cultured MSCs as pellets in a chondrogenic-differentiation medium supplemented with PL. After 21 days, the pellets were processed for histological analysis and stained with alician blue and acridine orange. The expression of *SOX9* was investigated using RT-PCR.

**Results:**

MSCs maintained their stemness characteristics in the PL-supplemented medium. However, the distribution of cells in the pellets cultured in the PL-supplemented chondrogenic differentiation medium had a greater similarity to cartilage tissue-derived chondrocytes than to the negative control. The intense alician blue staining indicated an increased production of mucopolysaccharides in the differentiated pellets, which also showed elevated expression of *SOX9*.

**Conclusions:**

Our data suggest that MSCs could be differentiated to chondrocytes in the presence of PL and absence of exogenous TGF-β. Further research needs to be conducted to understand the exact role and potential of PL in chondrogenic differentiation and chondrocyte regeneration.

## Introduction

Cartilage contains one type of cell, the chondrocytes, and lacks the capacity for self-regeneration [1]. Cells used to repair cartilage could be obtained by biopsies from uninjured cartilage, but this approach may cause in injury to healthy cartilage. There is an increasing effort to determine optimal protocols and conditions to direct the differentiation of stem cells into chondrocytes [1, 2].

Mesenchymal stem cells (MSCs) are capable of self-renewal, with the potential to differentiate into adipocytes, osteoblasts and chondrocytes, and possibly also into cardiomyocytes, skeletal myocytes, hepatocytes and neurons [3, 4]. They have been clinically investigated for their ability to repair cartilage [5]. They can be isolated and expanded from several sources, including the umbilical cord. Compared to other sources, the umbilical cord contains MSCs with higher proliferation potential and allows relatively easy and noninvasive isolation procedures [3, 4].

All detailed differentiation protocols use transforming growth factor-β (TGF-β) among other components in culture media to direct differentiation of MSCs to chondrocytes, because TGF-β signaling is crucial in chondrogenic differentiation [6, 7]. Recent protocols have focused on developing efficient chondrogenic differentiation conditions: environment, growth factors and types of culture system, including 3D-cultures [8, 9]. It is known that differentiation of MSCs is highly influenced by the surrounding niche or scaffold [10].

Prepared from platelet-enriched plasma, platelet lysate (PL) contains high concentrations of growth factors, including platelet-derived growth factor (PDGF), epidermal growth factor (EGF) and TGF-β, which all play important roles in stem cell growth, proliferation and differentiation [11, 12]. Several studies indicated that PL derived from apheresis concentrates successfully promotes MSCs proliferation with no significant differences compared to PL prepared from whole blood concentrates [13].

The reported methods to prepare PL include freeze–thaw (FT), sonication, and chemical or physiological stimulation [11, 14]. The quality is affected by number of factors, including preparation procedure, filtration steps and number of FT cycles. In fact, the FT method is simple, fast and effective compared to the chemical activation of platelets.

In the simple FT method, PL is produced by repeatedly freezing and thawing platelet-rich plasma (PRP) [11, 14], with three FT cycles proven to efficiently release a sufficient concentration of growth factors for MSCs proliferation that maintains immunophenotype and differentiation potency. Although there may be differences in growth factor concentrations in PL, depending on preparation method and individual platelet units, variations can be reduced by pooling platelet units [11, 13, 14].

Thanks to these properties, PL has been used as a substitute for fetal bovine serum (FBS) in MSCs culture and has been investigated for its ability to accelerate wound healing and support regeneration. A number of studies have also investigated the potential clinical benefits of PL in cartilage dysfunction [11, 15, 16].

Here, we investigate the ability of PL to act as a source of growth factors to induce differentiation of MSCs into chondrocytes. To achieve this, we used cell culture, histological and molecular methods.

## Materials and methods

### Preparation of platelet lysates and measurement of TGF-β content

Human platelet lysates (PL) were prepared from platelet-rich plasma (PRP) obtained from the Damascus University Blood Transfusion Center. PRP from 3 donors was pooled, frozen at − 80 °C and thawed at 37 °C. The FT cycle was repeated three times, then PL was centrifuged for 20 min at 15000 g and 4 °C, and filtered through 0.22-μm Ministart filters (Sartourius Stediumbiotech). Heparin (2 U/ml) (Syrbio) was added to filtered PL and aliquots were stored at − 20 °C until use. The content of TGF- β1 in PL samples was measured using the TGF- β1 ELISA kit (Sunred Bio).

### Culturing mesenchymal stem cells

MSCs were isolated from three umbilical cords obtained from healthy pregnancies during cesarean deliveries. Informed consent was received, according to guidelines of the Damascus University Ethical Committee.

To isolate the MSCs, we used the explant method, which relies on the MSCs ability to migrate from umbilical cord tissues and adhere to the surface of culture vessels without using digestive enzymes [17]. Umbilical cords (UCs) were cut into 5-cm^2^ segments that were further cut longitudinally to remove blood vessels. Afterwards, segments were transferred to 25-cm^2^ Primo TC Flask (Euroclone) and incubated at 37°C in a humidified atmosphere with 5% CO_2_ in DMEM (Euroclone) supplemented with 5% PL, 100 U/ml penicillin and 100 μg/ml streptomycin (Euroclone).

After 7 days, the medium was changed for the first time and later was changed every 3-4 days. On day 14, segments were removed and adherent cells were allowed to expand until they reached ~ 80% confluence. At this point, the cells were detached using trypsin and EDTA 0.05% (Euroclone), then centrifuged at 200 g for 5 min and subcultured by plating on 12-well plates at a density of 10,000 cells/well. This procedure was repeated until passage 3. The morphology of the MSCs was assessed using an Olympus IX50 inverted microscope.

### Immunophenotypic characterization of MSCs

Cells at passage 2 were washed with PBS and trypsinized as previously described. 5 × 10^5^ cells were suspended and stained with the following antibodies: CD105-PE (Invitrogen), CD90-FITC (Santa Cruz Biotechnology), CD44-PE (BioRad), CD34-PE (BD Bioscience) and CD45-FITC (Invitrogen). Cells were analyzed using a FACS-Calibur (Becton Dickinson) and the data were analyzed with CellQuest software (Becton Dickinson) and Flowing Software V2.5.1 (Turku Centre for Biotechnology).

### Adipogenic and osteogenic differentiation

MSCs at passage 2 were induced to differentiate into adipocytes using the hMSC Adipogenic Differentiation kit (Euroclone) and into osteocytes using the hMSC Osteogenic Differentiation kit (Euroclone) in 96-well plates. To detect the adipocytes and osteocytes, after 3 weeks of differentiation, the cells were fixed with 10% formaldehyde, and respectively stained with 2% sudan III for 5 min or 2% alizarin red (pH 4.2) for 20 min at room temperature. Lipid droplets and calcium deposits were detected using an Olympus IX50 inverted microscope.

### Chondrogenic differentiation

One million MSCs at passage 2 were centrifuged at 200 g for 5 min and the pellets were cultured in 15 ml conical tubes containing 1 ml of chondrogenic differentiation medium consisting of DMEM, 1× ITS, 100 nM dexamethasone, 50 mg/l ascorbic acid, and 5% PL. The medium was changed twice a week. After 3 weeks, chondrogenic differentiation was assessed via histochemical staining and RT-PCR. Pellets maintained in DMEM with 5% PL were used as negative controls and chicken articular cartilages were used as positive controls.

### Histochemical analysis of pellets

Pellets were fixed in 10% formaldehyde for 3 h at room temperature, dehydrated through a series of graded ethanol baths, and embedded in paraffin. Paraffin-embedded pellets were sectioned using a microtome (Microtec). Sections were deparaffinized in xylol and rehydrated through a series of graded ethanol baths, and further stained in 1% alcian blue (Carl Roth) for 30 min. For acridine orange staining, slides were incubated for 30 min in a 0.02 mg/ml acridine orange acetic solution (Carl Roth).

### Reverse transcription PCR (RT-PCR)

Total RNA was extracted from pellets at the end of week 3 using TRI Reagent (Sigma-Aldrich). Reverse transcription was carried out using a RevertAid First Strand Synthesis Kit (Thermo Fischer) and RT-PCR was performed using Dream Taq (Thermo Fischer) to detect the expression of the *SOX9* gene. The forward primer was 5’-ATCTGAAGAAGGAGAGCGAG-3′ and the reverse primer was 5’-TCAGAAGTCTCCAGAGCTTG-3′. *GAPDH* expression was used as a control with the forward primer 5’-CAAGGTCATCCATGACAACTTTG-3′ and reverse primer 5’-GTCCACCACCCTGTTGCTGTAG-3′. Amplicons were visualized on 2% agarose gel using a 100 pb ladder (GeneDireX) and ethidium bromide (Carl Roth). The gel image was analyzed and the relative expression calculated using Image J software (NIH).

## Results

### Mesenchymal stem cell culture and immunophenotyping

**Fig. 1:**
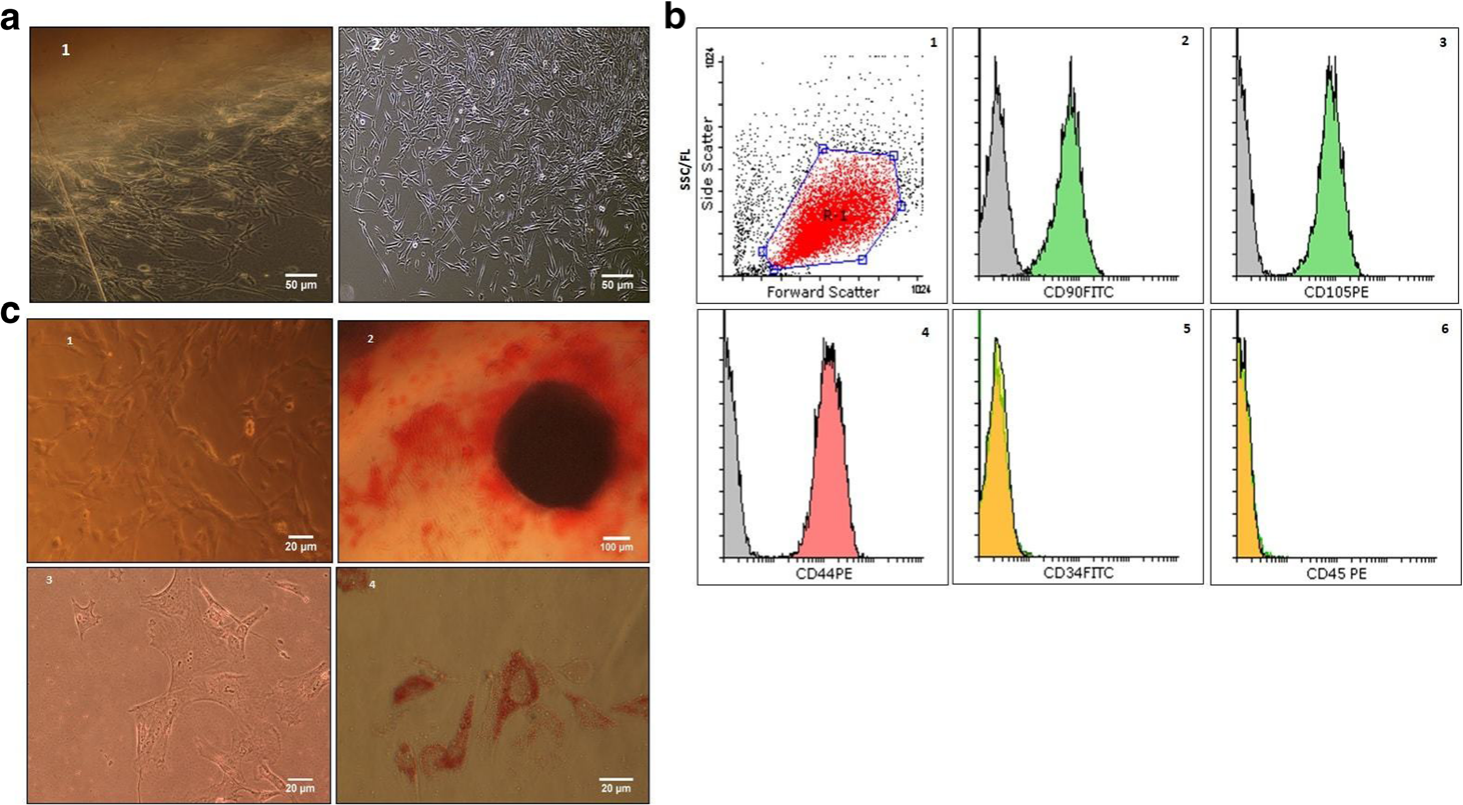
Morphology and characterization of MSCs cultured in medium supplemented with 5% PL. **a** Photos of cells at 150× magnification. i) Migrating cells appeared at the edge of the explants after 7 days. ii) Fibroblast-like morphology of MSCs in passage 3. **b** Flow cytometry of UCMSCs cultured in medium supplemented with 5% PL. FSC and SSC distribution of gated cells (i). The cells stained positive for CD90 (ii), CD105 (iii) and CD44 (iv), but negative for CD34 (v) and CD45 (vi). **c** Adipogenic and osteogenic differentiation of MSCs. Differentiated cells stained with alizarin red (i and ii) or sudan III (iii and iv). i and iii show the controls; ii shows positive for mineralization indicating osteogenic differentiation; iv shows positive staining for lipid vacuoles indicating adipogenic differentiation. The flow cytometry data are representative from one out of three donors tested (*n* = 3)

The MSCs isolated and expanded in the PL-supplemented medium showed a fibroblast-like morphology with multiple nucleoli (Fig. 1A). In the flow cytometry, they tested positive for CD90, CD105 and CD44 and negative for CD34 and CD45 (Fig. 1B).

### Adipogenic and osteogenic differentiation

The MSCs cultured in the PL-supplemented medium and directed to differentiate into osteocytes showed morphological changes and stained positive for alizarin red, indicating the presence of calcium deposits (Fig. 1C ii). The cells differentiated into adipocytes displayed an accumulation of lipid vacuoles that stained positive for sudan III (Fig. 1C iv). In the absence of adipogenic and osteogenic differentiation media, the control cells stained negative (Fig. 1C i and iii).

### Chondrogenic differentiation

**Fig. 2:**
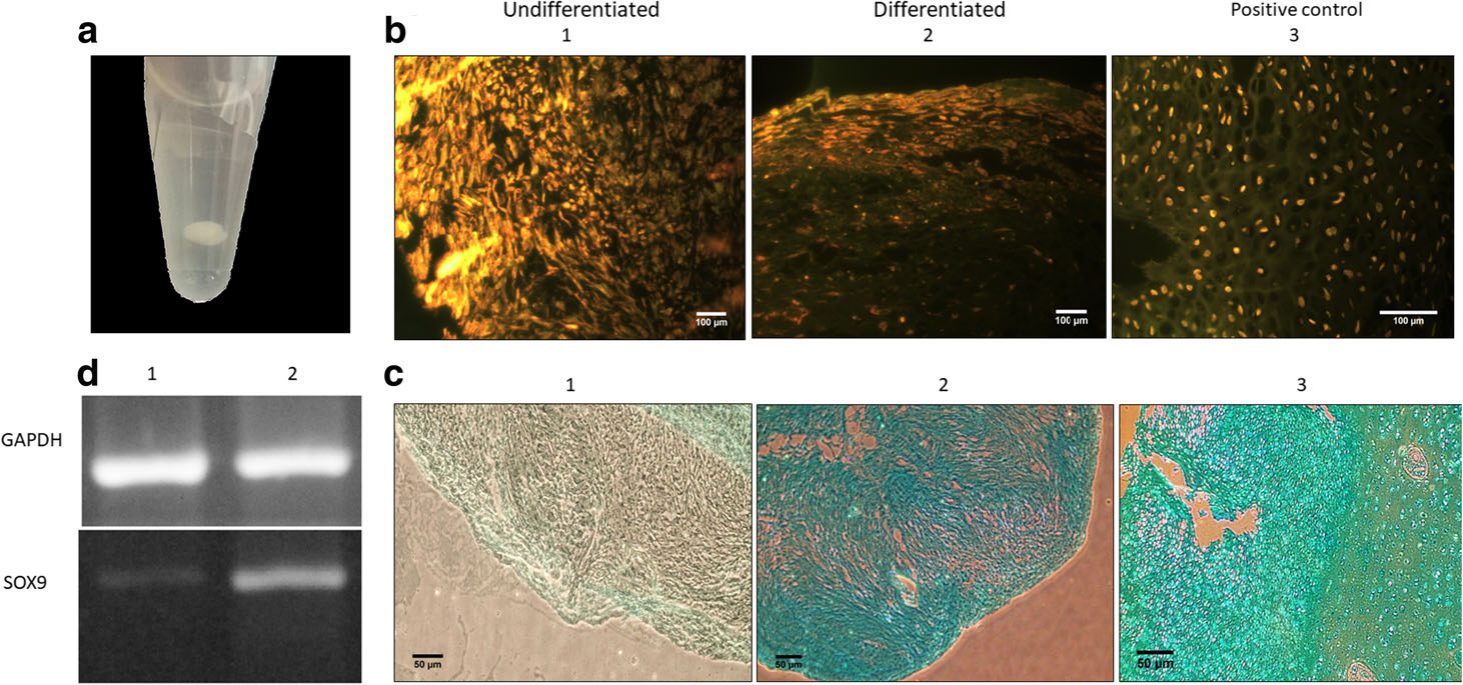
Characterization of differentiated MSCs pellets in chondrogenic differentiation medium supplemented with 5% PL. **a** Three-dimensional round tissue pellet after 3 weeks of culture in differentiation medium. **b** Acridine orange staining and the distribution of the cells in the pellets i) Sections of undifferentiated pellets (negative control), image at 150× magnification. ii) Sections of differentiated pellets, image at 150× magnification. iii) Sections of chicken articular cartilage (positive control), image at 600× magnification. **c** Alician blue staining. i) Sections of undifferentiated pellets (negative control), image at 150× magnification. ii) Sections of differentiated pellets, image at 150× magnification. iii) Sections of chicken articular cartilage, image at 300× magnification. **d** 2% agarose gel electrophoresis for amplified transcripts of *SOX9* and *GAPDH*. i) Amplified transcripts in undifferentiated pellets. ii) Amplified transcripts in differentiated pellets. The data are representative from one out of three donors tested (*n* = 3)

When pelleted, the MSCs cultured in chondrogenic differentiation medium supplemented with 5% PL formed solid three-dimensional round tissue balls, appearing as a mass at the bottom of the tube (Fig. 2a). ELISA showed that the average concentration of TGF-β1 in the PL was 153 ± 10 ng/ml, so the final concentration of TGF-β1 in the medium was close to 8 ng/ml.

Cell distribution was investigated using acridine orange staining. The distribution of cells in pellets cultured in chondrogenic differentiation medium was similar to that in chicken articular cartilage (positive control). Cells cultured in PL-supplemented medium without differentiation components (negative control) did not form any tissue structure reminiscent of cartilage (Fig. 2b). Alician blue staining revealed increased production of proteoglycan in cells cultured in chondrogenic differentiation medium supplemented with 5% PL compared with the negative controls. In fact, sections of pellets induced to chondrogenic differentiation showed dense blue staining close to the intensity of the positive controls (Fig. 2c).

### RT- PCR

RT-PCR products for *SOX9* and *GAPDH*, visualized on gel electrophoresis, indicated increased expression of *SOX9* in pellets cultured in chondrogenic differentiation medium supplemented with PL (Fig. 2d). The image analysis showed an elevated expression (~ 4.8 fold) of *SOX9* in differentiated compared to undifferentiated pellets, using *GAPDH* expression as a housekeeping gene.

## Discussion and conclusions

In this study, we investigated the possibility of using platelet lysate as a source of growth factors for inducing the differentiation of MSCs into chondrocytes. We cultured MSCs in a 3D system as pellets in chondrogenic differentiation medium supplemented with PL. Our results showed that culturing MSCs in a medium with 5% PL maintained the stemness characteristics of the MSCs, which is reflected by their morphology (Fig. 1A) and potential for differentiation into adipocytes and osteocytes (Fig. 1C).

Immunophenotyping of MSCs cultured in a medium with 5% PL revealed that these cells maintained standard positive and negative molecular profiles [18], indicating that the PL-supplement medium preserved the expression of MSCs surface markers during isolation and expansion periods (Fig. 1B).

Using PL as a xenogeneic-free and effective substitute for FBS in MSCs culture was also addressed in other studies, which showed that culturing MSCs in PL-supplemented media maintained both the immunophenotype profile and the differentiation potency of MSCs [13, 19–21]. However, previous studies did not focus on the possibility to use PL as a substitute for recombinant growth factors in chondrogenic differentiation of MSCs [13, 19–21].

In this study, we used PL in the chondrogenic differentiation medium without adding recombinant TGF-β, which is considered a basic and essential factor in chondrogenic differentiation medium [6]. Our results also showed that culturing MSCs in a 3D system as pellets in chondrogenic differentiation medium supplement with 5% PL, without the addition of purified recombinant TGF-β, generated a tissue structure similar to cartilage. In fact, acridine orange staining revealed a distribution of cells in pellets cultured in differentiation medium bearing a greater similarity to the distribution of chondrocytes in cartilage tissue (positive control) than the negative control (Fig. 2b).

It is noteworthy that the final concentration of TGF-β1 in our differentiation media was close to the concentration of recombinant protein added in standard chondrogenic differentiation protocols [2, 7].

Alician blue staining revealed that production of mucopolysaccharides and glycosaminoglycans was higher in pellets cultured in chondrogenic differentiation medium than in the negative control. The levels were close to those of the chicken articular cartilage sections (Fig. 2c).

At the molecular level, we showed that the expression of *SOX9* increased (~ 4.8 fold, measured semi-quantitatively) in pellets cultured in chondrogenic differentiation medium supplemented with 5% PL compared with pellets cultured in 5% PL-supplemented medium alone (Fig. 2d). *SOX9* is an early chondrogenic marker. The *SOX9* gene encodes a transcription factor with increased expression during chondrogenic differentiation and close correlation with the expression of collagen type II alpha 1 chain (*COL2A1*), the primary protein in the extracellular matrix of articular cartilage [22].

The increased expression of *SOX9* could be due to chondrogenic differentiation medium supplemented with 5% PL, which contains TGF-β and other growth factors, triggering chondrogenic differentiation, while 5% PL supplement medium without differentiation components only maintained MSCs stemness (Fig. 1A, B and C).

We showed that 5% PL-supplemented medium with other differentiation components, could direct the differentiation of MSCs to chondrocytes, which might be due to a high concentration of TGF-β in PL, since TGF-β is known to play a key role in the triggering of chondrocyte differentiation [6, 9].

The importance of our results relates to the potential clinical applications of PL in cartilage dysfunction. We showed that PL in the absence of TGF-β is capable of inducing both proliferation and chondrogenic differentiation of MSCs in vitro, which could further be extended to in vivo utility by co-injecting MSCs and PL into injured cartilage. This could be relevant for clinical application, since is avoids the possible side effects of exogenous TGF-β. Moreover, PL has been reported to have an anti-inflammatory and analgesic effect in some tissues, including cartilage. It may enhance the healing of injured cartilage, which it is easily attacked by proinflammatory factors and affected by oxidative stress [23, 24].

In conclusion, our results suggest that although MSCs maintain their stemness characteristics when cultured in a PL-supplemented medium, PL could also be used to induce chondrogenic differentiation of umbilical cord-derived MSCs in the absence of exogenous TGF-β. This differentiation would include increased expression of *SOX9* and increased mucopolysaccharide content. Further investigation should focus on comparing the effects of recombinant TGF-β with PL in triggering chondrogenic differentiation, assessing early and late chondrogenic markers, and on determining the optimal way to benefit from PL in cartilage regeneration in the clinical setup.

## Abbreviations

COL2A1: Collagen type II alpha 1 chain
DMEM: Dulbecco’s modified Eagle’s medium
EGF: Epidermal growth factor
FBS: Fetal bovine serum
*GAPDH*: Glyceraldehyde-3-phosphate dehydrogenase
MSCs: Mesenchymal stem cells
PDGF: Platelet-derived growth factor
PL: Platelet lysate
PRP: Platelet-rich plasma
RT-PCR: Reverse transcription polymerase chain reaction
*SOX-9*: SRY-Box 9
TGF-β: Transforming growth factor beta
UC: Umbilical cord

## References

[1] Vinatier C, Guicheux J (2016). Cartilage tissue engineering: from biomaterials and stem cells to osteoarthritis treatments. Ann Phys Rehabil Med.

[2] Solchaga LA, Penick KJ, Welter JF (2011). Chondrogenic differentiation of bone marrow-derived mesenchymal stem cells: tips and tricks. Methods Mol Biol.

[3] Can A, Karahuseyinoglu S (2007). Concise review: human umbilical cord stroma with regard to the source of fetus-derived stem cells. Stem Cells.

[4] Ding DC, Chang YH, Shyu WC, Lin SZ (2015). Human umbilical cord mesenchymal stem cells: a new era for stem cell therapy. Cell Transplant.

[5] Orth P, Rey-Rico A, Venkatesan JK, Madry H, Cucchiarini M (2014). Current perspectives in stem cell research for knee cartilage repair. Stem Cells Cloning.

[6] Tekari A, Luginbuehl R, Hofstetter W, Egli RJ (2015). Transforming growth factor beta signaling is essential for the autonomous formation of cartilage-like tissue by expanded chondrocytes. PLoS One.

[7] Mackay AM, Beck SC, Murphy JM, Barry FP, Chichester CO, Pittenger MF (1998). Chondrogenic differentiation of cultured human mesenchymal stem cells from marrow. Tissue Eng.

[8] Lee HJ, Choi BH, Min BH, Park SR (2009). Changes in surface markers of human mesenchymal stem cells during the chondrogenic differentiation and dedifferentiation processes in vitro. Arthritis Rheum.

[9] Moroz A, Bittencourt RA, Almeida RP, Felisbino SL, Deffune E (2013). Platelet lysate 3D scaffold supports mesenchymal stem cell chondrogenesis: an improved approach in cartilage tissue engineering. Platelets.

[10] Marrelli M, Falisi G, Apicella A, Apicella D, Amantea M, Cielo A, Bonanome L, Palmieri F, Santacroce L, Giannini S, Di Fabrizio E, Rastelli C, Gargari M, Cuda G, Paduano F, Tatullo M (2015). Behaviour of dental pulp stem cells on different types of innovative mesoporous and nanoporous silicon scaffolds with different functionalizations of the surfaces. J Biol Regul Homeost Agents.

[11] Bieback K (2013). Platelet lysate as replacement for fetal bovine serum in mesenchymal stromal cell cultures. Transfus Med Hemother.

[12] Mirabet V, Solves P, Minana MD, Encabo A, Carbonell-Uberos F, Blanquer A, Roig R (2008). Human platelet lysate enhances the proliferative activity of cultured human fibroblast-like cells from different tissues. Cell Tissue Bank.

[13] Fekete N, Gadelorge M, Fürst D, Maurer C, Dausend J, Fleury-Cappellesso S, Mailänder V, Lotfi R, Ignatius A, Sensebé L, Bourin P, Schrezenmeier H, Rojewski MT (2012). Platelet lysate from whole blood-derived pooled platelet concentrates and apheresis-derived platelet concentrates for the isolation and expansion of human bone marrow mesenchymal stromal cells: production process, content and identification of active components. Cytotherapy.

[14] Hemeda H, Giebel B, Wagner W (2014). Evaluation of human platelet lysate versus fetal bovine serum for culture of mesenchymal stromal cells. Cytotherapy.

[15] Yin W, Xu H, Sheng J, Xu Z, Xie X, Zhang C (2017). Comparative evaluation of the effects of plateletrich plasma formulations on extracellular matrix formation and the NFkappaB signaling pathway in human articular chondrocytes. Mol Med Rep.

[16] Tyrnenopoulou P, Diakakis N, Karayannopoulou M, Savvas I, Koliakos G (2016). Evaluation of intra-articular injection of autologous platelet lysate (PL) in horses with osteoarthritis of the distal interphalangeal joint. Vet Q.

[17] Hassan G, Kasem I, Soukkarieh C, Aljamali M (2017). A simple method to isolate and expand human umbilical cord derived mesenchymal stem cells: using explant method and umbilical cord blood serum. Int J Stem Cells.

[18] Dominici M, Le Blanc K, Mueller I, Slaper-Cortenbach I, Marini F, Krause D, Deans R, Keating A, Prockop D, Horwitz E (2006). Minimal criteria for defining multipotent mesenchymal stromal cells. The International Society for Cellular Therapy position statement. Cytotherapy.

[19] Shirzad N, Bordbar S, Goodarzi A, Mohammad M, Khosravani P, Sayahpour F, Baghaban Eslaminejad M, Ebrahimi M (2017). Umbilical cord blood platelet lysate as serum substitute in expansion of human mesenchymal stem cells. Cell Journal (Yakhteh).

[20] Astori G, Amati E, Bambi F, Bernardi M, Chieregato K, Schäfer R, Sella S, Rodeghiero F (2016). Platelet lysate as a substitute for animal serum for the ex-vivo expansion of mesenchymal stem/stromal cells: present and future. Stem Cell Res Ther.

[21] Antoninus AA, Widowati W, Wijaya L, Agustina D, Puradisastra S, Sumitro SB, Widodo MA, Bachtiar I (2015). Human platelet lysate enhances the proliferation of Wharton's jelly-derived mesenchymal stem cells. Biomarkers Genomic Med.

[22] Estes BT, Diekman BO, Gimble JM, Guilak F (2010). Isolation of adipose-derived stem cells and their induction to a chondrogenic phenotype. Nat Protoc.

[23] Zhang Y, Pizzute T, Pei M (2014). Anti-inflammatory strategies in cartilage repair. Tissue Eng Part B Rev.

[24] Forte D, Ciciarello M, Valerii MC, De Fazio L, Cavazza E, Giordano R, Parazzi V, Lazzari L, Laureti S, Rizzello F, Cavo M, Curti A, Lemoli RM, Spisni E, Catani L (2015). Human cord blood-derived platelet lysate enhances the therapeutic activity of adipose-derived mesenchymal stromal cells isolated from Crohn's disease patients in a mouse model of colitis. Stem Cell Res Ther.

